# Supplementary material for: Predicting visual function by interpreting a neuronal wiring diagram
Source: Nature. 2024 Oct 2;634(8032):113–23. doi: 10.1038/s41586-024-07953-5 (PMC11446822; doi:10.1038/s41586-024-07953-5)
Supplement: Supplementary file 6 — CRF and ERF predictions for individual TmY4 and TmY9 cells. Analogous to Supplementary Data 3, but for TmY target types. Shown are the top four monosynaptic pathways, the strongest pathway passing through each of the top ten intermediary types (ranking from Extended Data Fig. 7), and the trisynaptic pathway Tm1–TmY–Dm3–TmY (see the section entitled Prediction of spatial normalization). [file 41586_2024_7953_MOESM6_ESM.zip › DataS4/TmY9q/720575940629101135.pdf]

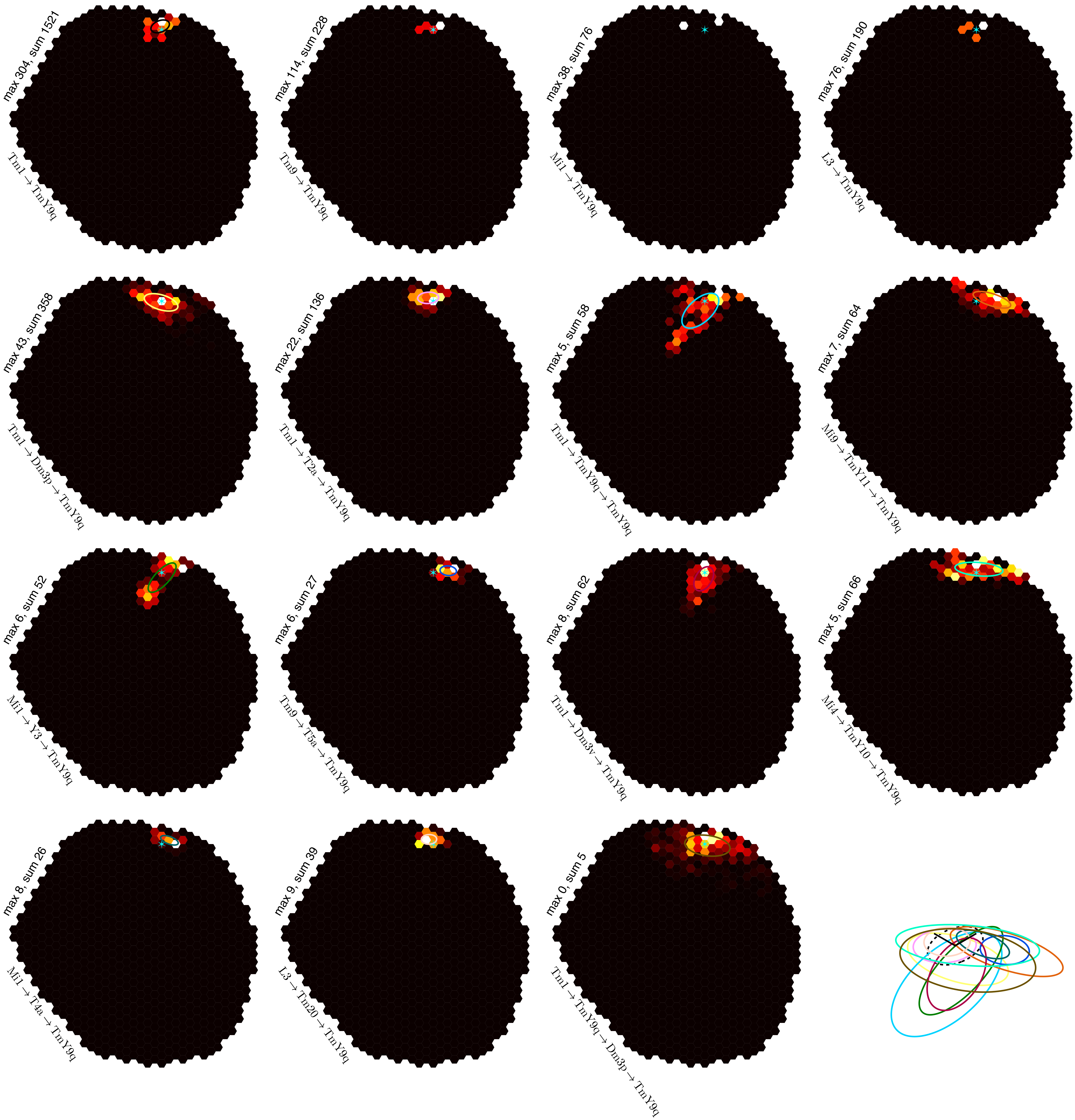
max 304, sum 1521
Tm1 → TmY9q
max 114, sum 228
Tm9 → TmY9q
max 38, sum 76
Mi1 → TmY9q
max 76, sum 190
L3 → TmY9q
max 43, sum 358
Tm1 → Dm3p → TmY9q
max 22, sum 136
Tm1 → T2a → TmY9q
max 5, sum 58
Tm1 → TmY9q → TmY9q
max 7, sum 64
Mi9 → TmY11 → TmY9q
max 6, sum 52
Mi1 → Y3 → TmY9q
max 6, sum 27
Tm9 → T5a → TmY9q
max 8, sum 62
Tm1 → Dm3v → TmY9q
max 5, sum 66
Mi4 → TmY10 → TmY9q
max 8, sum 26
Mi1 → T4a → TmY9q
max 9, sum 39
L3 → Tm20 → TmY9q
max 0, sum 5
Tm1 → TmY9q → Dm3p → TmY9q
